# Effect of hemoperfusion plus hemodialysis on uremic toxins and anemia in patients with maintenance hemodialysis

**DOI:** 10.1080/0886022X.2026.2642241

**Published:** 2026-03-30

**Authors:** Lu-Xi Zou, Xue Wang, Chen-Huan Qian, Xue-Qi Zheng, Ling Sun

**Affiliations:** a School of Management, Xuzhou Medical University, Xuzhou, Jiangsu, China; b Department of Nephrology, Xuzhou Clinical School of Xuzhou Medical University, Xuzhou, China; c Department of Nephrology, Xuzhou Central Hospital, Southeast University, Xuzhou, Jiangsu, China

**Keywords:** Hemodialysis, hemodiafiltration, hemoperfusion, uremic toxin, anemia

## Abstract

**Objectives: **This study aimed to compare the clearance of middle-molecular and protein-bound toxins among high-flux hemodialysis (HFHD), hemodiafiltration (HDF), and hemoperfusion combined with HD (HP-HD), and assess the impact of HP-HD on anemia management in patients with maintenance hemodialysis (MHD).

**Methods:** This prospective single-arm interventional study involved 39 stable MHD patients. We evaluated the serum levels of intact parathyroid hormone (iPTH), β2-microglobulin (β2-MG), hepcidin, hippuric acid (HA), indoxyl sulfate (IS), and p-cresyl sulfate (pCS) before and after individual sessions of HFHD, HDF, and HP-HD, separately. Linear mixed-effects model with Tukey-adjusted comparisons was applied to compare the effects of three modalities on intra-dialysis reduction ratios (RRs).

**Results:** Both HP-HD and HDF achieved greater RRs of iPTH, β2-MG, hepcidin, HA, and IS than HFHD (all *p* < 0.05), with no significant difference between HP-HD and HDF (all *p* > 0.05). Unlike the former five toxins, HP-HD performed better than HFHD in removing pCS (41.48 vs. 29.76%, *p* = 0.044), while HDF was not superior to HFHD (34.73 vs. 29.76%, *p* = 0.16). After the 6-month treatment with HP-HD weekly, the anemia and nutritional status were improved significantly, indicating by increasing hemoglobin (*p* = 0.006) and albumin (*p* = 0.043), and decreasing erythropoiesis-stimulating agent (ESA) resistance index (ERI) *(p* = 0.032).

**Conclusions:** Our findings suggested that the HP-HD might perform best in eliminating pCS, and HP-HD and HDF achieved equal clearances of middle-molecular toxins. The integrated blood purification protocol might improve anemia and nutritional status in MHD patients.

## Introduction

Chronic kidney disease (CKD), which may progress to end-stage renal disease (ESRD), is a major public health concern [1]. Maintenance hemodialysis (HD) serves as the cornerstone treatment for ESRD. However, conventional HD usually fails to remove uremic retention solutes [2], and these solutes accumulate mainly due to renal function decline in CKD patients [3].

Based on their physicochemical properties and the European Union Toxin Working Group (EUTox) classification, the uremic toxins are generally classified into three categories [2]: (1) Small water-soluble molecules (MW < 500 Da), including urea and creatinine, commonly used to assess dialysis adequacy. (2) Middle-large molecules (MW > 500 Da), such as β2-microglobulin and PTH, which are poorly cleared by conventional HD, but better removed with high-flux HD or hemodiafiltration (HDF). (3) Protein-bound molecules, such as indoxyl sulfate (IS) and p-cresyl sulfate (pCS), their removal is very limited even with high-flux HD and HDF, and the potential solution could be hemoperfusion (HP) with adsorbent systems [4,5].

Growing evidence demonstrated that the accumulated circulating uremic toxins were associated with anemia [6], inflammation [7], protein energy wasting [8], CKD progression [9], cardiovascular disease (CVD) and CVD-related mortality [10], overall mortality [11], as well as impaired cognitive function [12], and restless legs syndrome (RLS) [13]. The optimal integration of different dialysis modalities to adequately clear middle-large molecular and protein-bound uremic toxins, thereby reducing the complications in maintenance hemodialysis (MHD) patients, remains unclear. This study aimed to compare the effects of different dialysis modalities on the clearance efficiency of middle-large molecules and protein-bound toxins, as well as to assess their impact on anemia management, which may help inform practical dialysis prescription and improve the management of MHD patients.

## Materials and methods

### Study design and participants

We performed a prospective, single-arm interventional (self-control) study involving a cohort of stable MHD patients from the Blood Purification Center at Xuzhou Central Hospital (Xuzhou, China). Anemia management was conducted using erythropoiesis-stimulating agents (ESAs) and iron supplementation. Dosage adjustments were made monthly, in accordance with the Chinese clinical practice guideline [14]. Inclusion criteria consisted of: (1) ESRD diagnosed by estimated glomerular filtration rate (GFR) <15 mL/min/1.73m^2^ with chronic and irreversible kidney damage [15]; (2) age 18 years or older; (3) received HD treatment ≥ 3 months. Exclusion criteria consisted of: (1) overt inflammation/infection, or malignancy; (2) platelet levels < 100 × 10^9^/L or any coagulopathy; (3) cerebral or gastrointestinal hemorrhage within three months; (4) any hospitalization within three months; (5) unwillingness to provide written informed consent. The study was initiated in July 2023, with a 6-month follow-up period for all participants. All participants provided written informed consent, and the study protocol was approved by the ethical committee of the Xuzhou Central Hospital (Approval No. XZXY-LK-20230626-0101).

### Dialysis modalities

All patients were maintained on two high-flux hemodialysis (HFHD) and one online-HDF per week preceding the enrollment, then one HFHD session was replaced by HP combined with HFHD (HP-HD) once a week. HP was performed using KHA130 resin cartridge (Jafron Biomedical, China). The KHA130 cartridge, a Class III medical device (NMPA: 20193100932), utilizes neutral macroporous resin composed of double cross-linked styrene-divinylbenzene copolymer. HD was performed with high-flux dialyzers (e.g., B-16/18/20HF), and online-HDF was set as post-dilution using high-flux Dialyzers (e.g., FX80 and FX100) with surface area ranging from 1.6 to 2.0 m^2^ for 4 h per session (BFR = 250 ∼ 300 mL/min, DFR = 500 ∼ 800 mL/min, and post-dilution substitute fluid rate = 60 ∼ 90 mL/min). The dialysate and substitution fluid had an identical formulation across all patients: bicarbonate 32 mmol/L, sodium 140 mmol/L, potassium 2.0 mmol/L, calcium 1.5 mmol/L, magnesium 0.5 mmol/L, and glucose-free.

### Clinical data collection and measurements

Patient demographic and clinical data were recorded at baseline and six-month follow-up. For laboratory analysis, blood was collected at midweek immediately before dialysis and upon completion of each treatment session: HFHD, HDF, and HP-HD. All blood samples were collected midweek, with one-day gap, to minimize the impact of dialytic interval on the results. All post-dialysis samples were drawn from the arterial port of the dialysis circuit following a 2-min cessation of ultrafiltration. The clinical parameters, including intact parathyroid hormone (iPTH) and beta-2 microglobulin (β2-MG), were measured at an accredited lab (KingMed Diagnostics, China). The erythropoiesis-stimulating agent (ESA) resistance index (ERI) was calculated as: ERI = Weekly ESA Dose (IU)/[Hemoglobin (g/dL) × Body Weight (kg)]. Single-pool Kt/V (spKt/V) was determined using a two-point urea kinetic model that incorporated reductions in blood urea nitrogen and weight loss during a single dialysis session.

### Uremic toxins measurements

The quantitation of serum hippuric acid (HA), IS, and pCS was determined by high-performance liquid chromatography (HPLC) at a qualified lab (Guangdong Provincial Key Enterprise Laboratory for Medical Extracorporeal Circulation Adsorption and Separation Technology, Shenzhen, China). Since the target analytes (HA, IS, pCS) existed in free and protein-bound forms in the samples, a protein denaturation step was incorporated to liberate the bound fraction, ensuring the detection reflected the total concentration of each toxin. Quantification of serum hepcidin was performed using competitive ELISA kits (Cat. CSB-E13062h, Cusabio, China), which demonstrated good reproducibility, with intra- and inter-assay coefficients of variation (CV) both less than 15%.

The reduction ratio (RR) of uremic toxins was calculated using the following formula: RR = [(Cpre – Cpost)/Cpre] × 100%, where Cpre and Cpost represent serum concentrations before and after one single dialysis session, respectively. The Cpre and Cpost values of uremic toxins were adjusted by total serum protein concentration at Cpre and Cpost. Therefore, a positive value denoted a reduction in serum concentration within a single treatment session.

### Statistical analysis

Baseline and follow-up characteristics were described using proportions, medians [interquartile range (IQR)], or mean (±SD). For comparative analyses, paired t-test was applied to normally distributed data, while Wilcoxon matched-pairs signed-rank test was employed for non-normally distributed variables. The effects of dialysis modality on toxin reduction ratio (RR) were evaluated using a linear mixed-effects model, with Tukey-adjusted comparisons. A two-sided *p*-value <0.05 was considered statistically significant. Statistical analysis was performed using the statistical analysis system SAS 9.4 (SAS Institute of North Carolina, USA) and the R language (version 4.3).

## Results

### Effect of dialysis modalities on the clearance of uremic toxins

Thirty-nine MHD patients (female = 23, male = 16) participated and completed this study, with a median age of 58 years (range 33–90) at the baseline (Table 1). The spKt/V values had no significant differences across the three modalities (all *p* > 0.05) (Table S1).

**Table 1.** Baseline and 6-month follow-up characteristics of the MHD patients.

Variables	Baseline (*n* = 39)	6-Month follow-up (*n* = 39)	*p*-Value
Female	23 (59.0%)	23 (59.0%)	/
Age (years)	58.00 [45.50–65.50]	58.50 [46.00–66.00]	/
Diabetes mellitus	24 (61.5%)	24 (61.5%)	/
Dialysis vintage (month)	55 [35–84]	61 [41–90]	/
Hemoglobin (g/L)	108 [95–117]	115 [106–121]	0.003
Hematocrit (%)	32.40 [29.45–35.55]	35.10 [32.55–37.50]	0.007
ERI (U/kg/week/g/L)	11.28 [5.21–15.89]	5.30 [4.01–12.37]	0.005
White Blood Cells (×10⁹/L)	5.97 [4.78–7.33]	6.35 [4.85–7.43]	0.143
Neutrophil Percentage (%)	68.90 [65.09–73.28]	69.71 [64.20–74.40]	0.279
Platelets (×10⁹/L)	170 [124–216]	182 [129–223]	0.064
hs-CRP (mg/L)	4.36 [2.18–7.22]	3.23 [1.97–5.91]	0.035
Albumin (g/L)	40.00 [38.30–41.75]	41.60 [39.00–44.92]	<0.001
Total Cholesterol (mmol/L)	3.72 [2.84–4.09]	3.68 [3.09–4.22]	0.195
HDL-C (mmol/L)	0.84 [0.76–0.91]	0.84 [0.71–0.95]	0.224
LDL-C (mmol/L)	2.20 [1.60–2.54]	2.16 [1.66–2.58]	0.432
Pre-dialysis BUN (mmol/L)	27.16 [20.91–31.36]	25.49 [19.99–29.69]	0.239
Creatinine (μmol/L)	1093 [832.50–1261.50]	919 [757.50–1209.50]	0.286
Uric Acid (μmol/L)	442 [394–502]	394 [231–483]	0.002
Ferritin (μg/L)	166.60 [91.03–384.69]	188.20 [56.69–292.93]	0.124
Iron (μmol/L)	12.20 [10.20–15.85]	11.50 [9.50–13.45]	0.224
UIBC (μmol/L)	35.60 [26.45–43.15]	36.50 [30.70–46.20]	0.304
TIBC (μmol/L)	47.50 [40.40–55.10]	47.50 [42.90–56.70]	0.542
TSAT (%)	26.00 [20.78–34.67]	28.04 [19.00–33.13]	0.307
Vitamin B12 (pmol/L)	1104 [550–2000]	1002 [543–2000]	0.469
Folate (nmol/L)	4.40 [3.30–7.53]	5.80 [4.52–20.00]	0.006
Calcium (mmol/L)	2.17 [2.08–2.29]	2.26 [2.19–2.38]	0.04
Phosphorus (mmol/L)	2.44 [1.99–2.70]	1.82 [1.15–2.46]	0.002
Magnesium (mmol/L)	1.16 [1.08–1.26]	1.09 [1.02–1.18]	0.002
TCO₂ (mmol/L)	19.60 [16.26–21.35]	19.40 [17.09–22.75]	0.134
iPTH (pg/ml)	350.90 [247.00–488.47]	333.26 [232.16–456.31]	<0.001
β2-MG (mg/L)	39.99 [35.63–48.13]	37.14 [33.92–46.77]	<0.001
Hepcidin (ng/ml)	111.08 [57.56–248.82]	113.12 [54.41–193.18]	0.243
Hippuric acid (mg/L)	35.42 [19.45–57.84]	23.73 [12.91–33.60]	0.001
Indoxyl sulfate (mg/L)	45.22 [29.14–61.84]	40.09 [21.43–57.29]	0.229
p-cresyl sulfate (mg/L)	33.50 [19.80–44.92]	22.84 [13.25–42.94]	0.011

*Abbreviations:* MHD, maintenance hemodialysis; ERI, erythropoiesis stimulating agents (ESA) resistance index; hs-CRP, high sensitivity C-reactive protein; HDL-C, high-density lipoprotein cholesterol; LDL-C, low-density lipoprotein cholesterol; BUN, blood urea nitrogen; UIBC, unsaturated iron-binding capacity; TIBC, total iron-binding capacity; TSAT, transferrin saturation; TCO_2_, total carbon dioxide; iPTH, intact parathyroid hormone; β2-MG, β2-microglobulin

**Figure 1.**
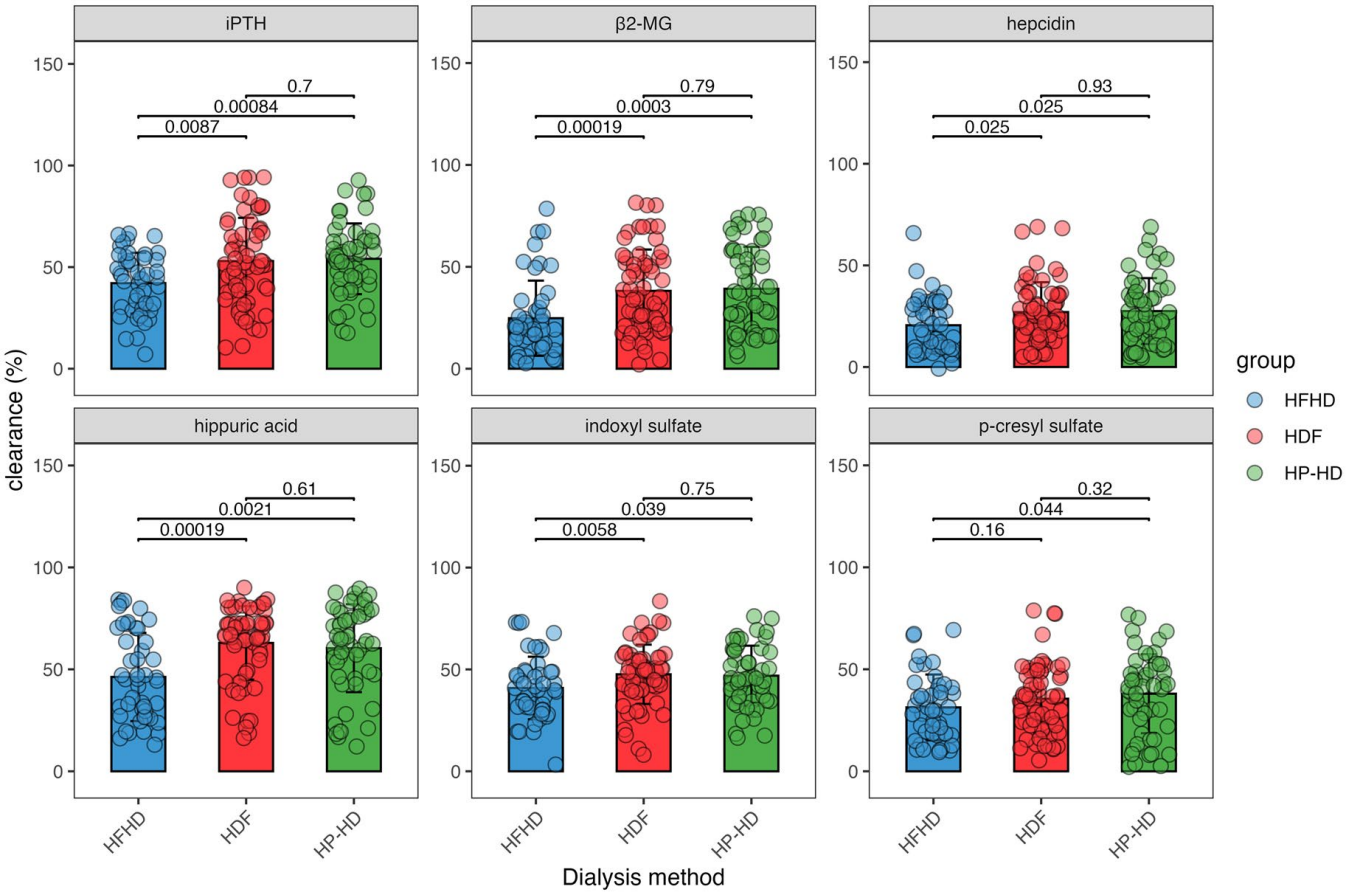
Comparison of the reduction ratio of iPTH (A), β2-MG (B), hepcidin (C), hippuric acid (D), indoxyl sulfate (E), and p-cresyl sulfate (F) during one dialysis session.

For the efficacy in removal of middle molecules, compared with HFHD, the RR values of iPTH were significantly higher in HDF (51.89 vs. 44.29%, *p* = 0.0087) and HP-HD (55.54 vs. 44.29%, *p* = 0.00084) sessions, while there was no difference between HDF and HP-HD (51.89 vs. 55.54%, *p* = 0.7). Similarly, the RR values of β2-MG were significantly higher in HDF (34.73 vs. 19.64%, *p* = 0.00019) and HP-HD (37.6 vs. 19.64%, *p* = 0.0003) than the HFHD session, and no significant difference was observed when comparing HDF with HP-HD (34.73 vs. 37.6%, *p* = 0.79) (Figure 1, Table 2, and Table S1).

For the protein-bound uremic toxins, both HDF and HP-HD sessions showed significantly higher RR values of hepcidin, HA, and IS compared to HFHD, with all *p*-values < 0.05. Meanwhile, no significant difference was observed between the HDF and HP-HD sessions (all *p*-values > 0.05). The clearance of pCS in the HP-HD session was higher than the HFHD session (41.48 vs. 29.76%, *p* = 0.044); however, HDF was not superior to HFHD in removing pCS during one dialysis session (34.73 vs. 29.76%, *p* = 0.16) (Figure 1, Table 2, and Table S1).

**Table 2.** Effect of three dialysis modalities on the reduction ratio (RR) of uremic toxins.

Variables	HFHD	HDF	HP-HD	*p*-Value
spKt/V	1.34 (1.25–1.38)	1.37 (1.25–1.47)	1.36 (1.28–1.42)	0.611
Pre-dialysis albumin (g/L)	39.6 (37.81–41.09)	39.39 (37-40.3)	39.5 (37.6–40.4)	0.077
Post-dialysis albumin (g/L)	41.81 (41-45.83)	43.48 (40.59–47.32)	41.76 (40.44–45.87)	0.078
Pre-dialysis iPTH (pg/ml)	321.83 (259.74–563.53)	421.19 (271.76–568.2)	392.95 (268.28–547.57)	0.989
Post-dialysis iPTH (pg/ml)	258.82 (121.98–312.99)	273.54 (60.89–306.55)	179.2 (107.25-301)	0.174
iPTH RR (%)	44.29 (29.92–53.97)	51.89 (38.41–67.25)	55.54 (44.31–62.91)	0.002
Pre-dialysis β2-MG (mg/L)	39.71 (37.3–46.8)	39.99 (37.44–46.8)	42.89 (36.77–47.95)	0.837
Post-dialysis β2-MG (mg/L)	34.62 (33.17–35.58)	29.29 (20.68–34.82)	28.56 (20.76–34.77)	0.001
β2-MG RR (%)	19.64 (13.13–29.09)	34.73 (21.7–52.41)	37.6 (20.21–57.7)	<0.001
Pre-dialysis hepcidin (ng/ml)	87.39 (65.2–243.69)	101.4 (73.58–169.22)	84.02 (35.02–176.21)	0.222
Post-dialysis hepcidin (ng/ml)	83.24 (59.33–233.32)	85.34 (56.88–132.48)	73.68 (23.13–146.78)	0.150
Hepcidin RR (%)	18.29 (9.22–31.31)	25.02 (17.1–35.32)	26.21 (14.42–36.81)	0.003
Pre-dialysis HA (mg/L)	26.45 (8.82–51.29)	42.73 (23.52–70.36)	36.73 (20.21–59.03)	0.019
Post-dialysis HA (mg/L)	14.38 (6.43–18.05)	17.27 (9.16–20.74)	13.05 (7.32–20.05)	0.250
HA RR (%)	45.14 (27-66.81)	66.61 (56.76–72.65)	64.64 (47.83–77.43)	<0.001
Pre-dialysis IS (mg/L)	43.43 (28.81–69.22)	39.67 (22.02–67.93)	44.33 (27.63–62.63)	0.859
Post-dialysis IS (mg/L)	23.89 (15.19–44.62)	22.44 (14.23–34.03)	22.45 (16.18–29.78)	0.165
IS RR (%)	39.9 (30.79–48.77)	49.36 (41.06–55.31)	45.65 (36.15–59.45)	0.026
Pre-dialysis pCS (mg/L)	35.93 (19.58–47.81)	30.35 (16.71–48.13)	32.01 (19.59–44.92)	0.049
Post-dialysis pCS (mg/L)	22.86 (14.31–29.37)	20.38 (11.92–30.87)	19.58 (11.44–25.27)	0.021
pCS RR (%)	29.76 (18.47–40.33)	36.51 (22.7–48.81)	41.48 (26.41–49.41)	0.089

*Abbreviations:* RR, reduction ratio; HFHD, high-flux hemodialysis; HDF, hemodiafiltration; HP-HD, hemoperfusion combined with high-flux hemodialysis; spKt/V, single-pool Kt/V; iPTH, intact parathyroid hormone; β2-MG, β2-microglobulin; HA, hippuric acid; IS, indoxyl sulfate; pCS, p-cresyl sulfate

### Effect of weekly HP-HD on anemia and serum levels of uremic toxins

After 6-month treatment with HP-HD once a week, there was a significant increase in hemoglobin and hematocrit (HCT), a decrease in ERI (all *p* < 0.05), indicating that anemia and ESAs responsiveness were improved. Meanwhile, there was a significant increase in serum albumin (*p* < 0.001) and folate (*p* = 0.005), alongside a significant decrease in hs-CRP (*p* = 0.035), suggesting an improvement in both inflammatory and nutritional status. Moreover, serum phosphorus demonstrated a significant decrease (*p* = 0.002), with serum slightly increasing (*p* = 0.04), which was indicative of improved chronic kidney disease-mineral and bone disorder (CKD-MBD). Furthermore, the pre-dialysis serum iPTH decreased from 350.90 to 333.26 pg/mL (*p* < 0.001), serum β2-MG decreased from 39.99 to 37.14 mg/L (*p* < 0.001), serum HA decreased from 35.42 to 23.73 mg/L (*p* = 0.001), and serum pCS decreased from 33.5 to 22.84 mg/L (*p* = 0.011). Meanwhile, pre-dialysis serum IsS only presents a decreasing trend without a significant statistical difference (*p* = 0.229) (Table 1).

However, the 6-month HP-HD treatment resulted in no statistically significant changes in iron metabolism parameters (serum iron, ferritin, UIBC, TIBC, and TSAT). Serum hepcidin levels did not change significantly over the course of the study, as summarized in Table 1.

## Discussion

At present, there are three modes of blood purification: conventional HD, HDF, and HP, of which HD could remove small-molecule toxins effectively; HDF is superior to HD in removing middle-molecule toxins [16,17]; HP is usually performed in combination with HD (HP-HD) to enhance elimination of medium-large molecules and protein-bound toxins by adding adsorption to diffusive/convective clearance [4,18].

PTH, a central mediator in CKD-mineral and bone disorder (CKD-MBD), is classified as a middle-molecule uremic toxin (MW 9.4 kDa) by the EUTox. In clinical practice, serum levels of iPTH are influenced more by serum phosphate and pharmacological therapy (e.g., vitamin D and calcimimetics) than by dialytic elimination [17]. β2-MG (MW 11.8 kDa), a key element for major histocompatibility antigen and dialysis-related amyloidosis, usually serves as a biomarker for evaluating middle-molecules retention and dialytic removal in MHD patients [17,19]. Our results showed that HDF and HP-HD exhibited comparable efficiencies in removing iPTH and β2-MG, and both performed significantly better than high-flux HD during one dialysis session.

Hepcidin (MW 2.8 kDa) is a key regulator of iron homeostasis by controlling cellular iron release. Its serum level could be increased by iron overload, infection/inflammation, and impaired renal function, and decreased by ESA administration and erythropoietic stimulation [20,21]. Hepcidin exhibited an ability to bind with plasma proteins, with binding affinities ranging from 3% to 89%, with large individual variability [22]. Considering the reduced renal clearance of hepcidin in ESRD patients, prolonged elevation of serum hepcidin levels may enhance its binding to proteins over time. Therefore, hepcidin should be classified as a middle-molecular protein-bound uremic toxin. Our previous study reported that incorporating HP into HD/HDF significantly enhanced hepcidin clearance over that achieved by HD/HDF modalities alone [23]. This study showed that the removal efficiency of hepcidin was comparable between HDF and HP-HD, with both exhibiting a significant advantage over HFHD in a single treatment.

Conventional HD and HDF usually fail to eliminate protein-bound uremic toxins, contributing to their accumulation in MHD patients [19]. Based on the affinity for protein binding, these toxins could be classified into three types: low protein binding affinity (e.g., HA), medium protein binding affinity (e.g., IS and pCS), and high protein binding affinity [e.g., 3-Carboxy-4-methyl-5-propyl-2-furalpropionic acid (CMPF)], of which the protein binding rate of CMPF is nearly 100%. Previous study had demonstrated that HP performed the best in removing CMPF, and HFHD was completely unable to remove it [5]. Thus, this study compared the effects of three dialysis modalities on the clearance of HA (MW 179 Da), IS (MW 188 Da), and pCS (MW 213 Da). Our results displayed that the clearance of pCS was superior in HP-HD compared to HFHD, but HDF did not surpass HFHD within a single dialysis session. Meanwhile, HDF and HP-HD had higher clearance of HA and IS than HFHD, with no difference between HDF and HP-HD.

Few studies have reported the impact of different dialysis modes on anemia management. In this study, we added HP-HD to conventional HD/HDF once a week for the MHD participants and followed up for 6 months. Our results showed that their anemia and nutritional status were significantly improved, manifested as hemoglobin and albumin increased, and ERI decreased. The mechanism may be related to more effective removal of middle-large molecular and protein-bound uremic toxins, with a significant decrease of serum iPTH, β2-MG, HA, and pCS from the baseline to 6-month follow-up. However, the pre-dialysis serum levels of hepcidin and IS did not show significant decreases; a possible explanation was that the timing of blood sampling may influence toxin levels. For instance, the serum levels of toxins might rebound to baseline by several days after dialysis treatment; therefore, weekly HP-HD could not lead to a reduction in pre-dialysis serum levels, while its time average concentration (TAC) was inferior to conventional dialysis treatment, which also resulted in a reduction in long-term and cumulative exposure risks [7].

Besides dialysis removal, there were multiple strategies to reduce the circulating levels of protein-bound uremic toxins. Increasing the intake of dietary fiber (e.g., high-amylose maize resistant starch type 2, HAM-RS2) could lead to a decrease in circulating gut-derived solutes, such as IS and pCS [24,25], and dietary fiber might also improve anemia by regulating the gut microbiota and short-chain fatty acids (SCFAs) in ESRD patients [26]. The administration of probiotics, prebiotics, and synbiotics was effective in reducing serum IS, pCS, urea, and creatinine through gut microbiota modulation [27]. Ramadan fasting could lead to a notable decrease in bacterial endotoxins and markers of inflammation in MHD patients [28]. In clinical practice, we should adopt a comprehensive management approach to reduce the levels of uremic toxins in MHD patients.

This study had strengths. Previous studies demonstrated that HP-HD could reduce the CVD mortality [29], improve inflammation [7], nutritional status and living quality [8], as well as prolong life expectancy [30], and the cost-effectiveness analysis supported the employment of HP-HD for ESRD patients in China [31]. Our findings revealed that combined HP with HD might help to improve anemia management through enhancing the clearance of middle-large molecular and protein-bound uremic toxins in MHD patients.

There were limitations. This study was conducted at a single center raises the possibility of bias in patient selection. Anemia significantly improved in MHD patients during follow-up, potentially driven by the enhanced clearance of uremic toxins in HP-HD sessions, which reduced the TAC and cumulative exposure risks of these toxins; however, the precise underlying mechanism remains to be elucidated.

In conclusion, the HP-HD might achieve the best clearance of pCS among the three dialysis modalities. HP-HD and HDF performed equally in removing iPTH, β2-MG, hepcidin, HA, and IS; both were superior to HFHD. The combined dialysis modes could improve anemia and nutritional status in MHD patients; the exact mechanism remains unclear and warrants validation in larger, longer-term cohorts.

## Supplementary Material

Supplemental Material

## Data Availability

The data are currently not publicly available due to participant privacy, but they are available from the corresponding author upon reasonable request.
