# Supplementary material for: Effect of hemoperfusion plus hemodialysis on uremic toxins and anemia in patients with maintenance hemodialysis
Source: Ren Fail. 2026 Mar 30;48(1):2642241. doi: 10.1080/0886022X.2026.2642241 (PMC13037201; doi:10.1080/0886022X.2026.2642241)
Supplement: Supplemental Material [file IRNF_A_2642241_SM6586.docx]

**Table S1. Pairwise comparisons for all outcomes**

| **Variables** | **P(HFHD vs HDF)** | **P(HFHD vs HP-HD)** | **P(HDF vs HP-HD)** |
| --- | --- | --- | --- |
| **spKt/V** | 0.29949 | 0.18459 | 0.7928 |
| **Pre-dialysis albumin (g/L)** | 0.3817 | 0.60704 | 0.7294 |
| **Post-dialysis albumin (g/L)** | 0.97618 | 0.64815 | 0.6172 |
| **Pre-dialysis iPTH (pg/ml)** | 0.40816 | 0.58346 | 0.8057 |
| **Post-dialysis iPTH (pg/ml)** | 0.2758 | 0.25382 | 0.9354 |
| **iPTH RR (%)** | 0.00872 | < 0.001 | 0.7033 |
| **Pre-dialysis β2-MG (mg/L)** | 0.91678 | 0.82219 | 0.8184 |
| **Post-dialysis β2-MG (mg/L)** | 0.00405 | 0.00158 | 0.9054 |
| **β2-MG RR (%)** | < 0.001 | < 0.001 | 0.785 |
| **Pre-dialysis hepcidin (ng/ml)** | 0.65228 | 0.11266 | 0.3377 |
| **Post-dialysis hepcidin (ng/ml)** | 0.34416 | 0.09329 | 0.3934 |
| **Hepcidin RR (%)** | 0.02502 | 0.02532 | 0.929 |
| **Pre-dialysis HA (mg/L)** | 0.00401 | 0.07004 | 0.2378 |
| **Post-dialysis HA (mg/L)** | 0.11365 | 0.67281 | 0.0646 |
| **HA RR (%)** | < 0.001 | 0.00211 | 0.606 |
| **Pre-dialysis IS (mg/L)** | 0.70244 | 0.8675 | 0.7417 |
| **Post-dialysis IS (mg/L)** | 0.28667 | 0.41193 | 0.8627 |
| **IS RR (%)** | 0.00578 | 0.03944 | 0.7479 |
| **Pre-dialysis pCS (mg/L)** | 0.99524 | 0.4454 | 0.469 |
| **Post-dialysis pCS (mg/L)** | 0.51915 | 0.0937 | 0.2303 |
| **pCS RR (%)** | 0.15987 | 0.04423 | 0.319 |

Abbreviations: RR, reduction ratio; HFHD, high-flux hemodialysis; HDF, hemodiafiltration; HP-HD, hemoperfusion combined with high-flux hemodialysis; spKt/V, single-pool Kt/V; iPTH, intact parathyroid hormone; β2-MG, β2-microglobulin; HA, hippuric acid; IS, indoxyl sulfate; pCS, p-cresyl sulfate.
